# Mapping Quantitative Trait Loci of Resistance to Tomato Spotted Wilt Virus and Leaf Spots in a Recombinant Inbred Line Population of Peanut (*Arachis hypogaea* L.) from SunOleic 97R and NC94022

**DOI:** 10.1371/journal.pone.0158452

**Published:** 2016-07-18

**Authors:** Pawan Khera, Manish K. Pandey, Hui Wang, Suping Feng, Lixian Qiao, Albert K. Culbreath, Sandip Kale, Jianping Wang, C. Corley Holbrook, Weijian Zhuang, Rajeev K. Varshney, Baozhu Guo

**Affiliations:** 1 USDA-ARS, Crop Protection and Management Research Unit, Tifton, United States of America; 2 International Crops Research Institute for the Semi-Arid Tropics (ICRISAT), Hyderabad, India; 3 The University of Georgia, Department of Plant Pathology, Tifton, United States of America; 4 College of Tropical Biology and Agronomy, Hainan Tropical Marine University, Sanya, China; 5 College of Life Science, Qingdao Agricultural University, Qingdao, Shandong, China; 6 The University of Florida, Department of Agronomy, Gainesville, United States of America; 7 USDA-ARS, Crop Genetics and Breeding Research Unit, Tifton, United States of America; 8 Fujian Agricultural and Forestry University, College of Plant Protection, Fuzhou, China; New Mexico State University, UNITED STATES

## Abstract

Peanut is vulnerable to a range of diseases, such as Tomato spotted wilt virus (TSWV) and leaf spots which will cause significant yield loss. The most sustainable, economical and eco-friendly solution for managing peanut diseases is development of improved cultivars with high level of resistance. We developed a recombinant inbred line population from the cross between SunOleic 97R and NC94022, named as the S-population. An improved genetic linkage map was developed for the S-population with 248 marker loci and a marker density of 5.7 cM/loci. This genetic map was also compared with the physical map of diploid progenitors of tetraploid peanut, resulting in an overall co-linearity of about 60% with the average co-linearity of 68% for the A sub-genome and 47% for the B sub-genome. The analysis using the improved genetic map and multi-season (2010–2013) phenotypic data resulted in the identification of 48 quantitative trait loci (QTLs) with phenotypic variance explained (PVE) from 3.88 to 29.14%. Of the 48 QTLs, six QTLs were identified for resistance to TSWV, 22 QTLs for early leaf spot (ELS) and 20 QTLs for late leaf spot (LLS), which included four, six, and six major QTLs (PVE larger than 10%) for each disease, respectively. A total of six major genomic regions (MGR) were found to have QTLs controlling more than one disease resistance. The identified QTLs and resistance gene-rich MGRs will facilitate further discovery of resistance genes and development of molecular markers for these important diseases.

## Introduction

Peanut or groundnut (*Arachis hypogaea* L.) is cultivated in more than 100 countries and serves as a major source of nutrition. This crop is cultivated on 25.7 million hectares with a global production of 42.3 million tons during 2014 [1]. The major producing countries are China, India, Nigeria, and USA. Peanut also serves as forage for animals, thus, providing additional revenue to farmers. The nitrogen fixing ability of peanut helps in enhancing soil fertility and thus further improves financial return for farmers. However, peanut is also susceptible to many biotic and abiotic stresses affecting peanut production worldwide. Genomic technology should aid peanut breeding programs in developing high yielding cultivars with improved stress tolerance. The important biotic stresses of peanut include Tomato spotted wilt virus (TSWV) caused by a tospovirus (family *Bunyaviridae*; genus *Tospovirus*) transmitted by thrips, early leaf spot (ELS) caused by *Cercospora arachidicola* and late leaf spot (LLS) caused by *Cercosporidium personatum* affecting adversely the peanut productivity and quality. For instance, the TSWV disease is more severe in peanut production areas in the southern US with an average loss of US$ 12.3 million annually (1996 to 2006) in the US [2]. Similarly, the leaf spots diseases (both early and late) are major diseases in peanut growing regions across the world causing yield loss up to 70% and economic losses around US$ 599 million globally [1, 3–6].

Although these diseases can be controlled by spraying chemicals such as insecticides and fungicides, this increases production costs for farmers and also pollutes the environment. Therefore, the most economical and sustainable solution is to breed disease resistant cultivars with high yield [7–10]. Despite being limited success, majority of the peanut cultivar development programs across world use conventional breeding methods for breeding new peanut cultivars. The application of new biotechnology such as molecular marker-assisted selection (MAS) in breeding programs has been shown to increase the genetic gains significantly per selection cycle when compared to that of conventional breeding [9]. The major obstacles hindering the application of MAS in peanut are the very narrow genetic diversity and limited numbers of polymorphic DNA markers. The development of a dense genetic map and the use of the map to identify the genes/markers should enable more MAS breeding efforts in peanut genetic improvement programs.

During the past decade, there have been significant advancements in high throughput genotyping platforms, next-generation sequencing technologies, trait mapping approaches and genomics-assisted breeding (GAB) [9–10]. Recent literature clearly suggests that high throughput genotyping and multi-season/location phenotyping data will facilitate high resolution and accurate marker-trait associations leading to development of diagnostic markers for desirable traits. Further, these diagnostic markers together with other genome-wide markers can be deployed effectively to more rapidly achieve higher genetic gains. Advanced genomic resources have already been deployed at a large scale for several trait mapping and breeding applications in peanut [7–10].

Several studies on trait mapping either through genetic mapping or linkage disequilibrium (LD) mapping approaches have been conducted in last five years. None of these studies targeted ELS and TSWV disease except earlier study conducted using the same population using sparse genetic map. Nevertheless, Khedikar et al. [11] and Sujay et al. [12] have identified major QTLs for LLS using the RIL population (TAG 24 × GPBD 4) where GPBD 4 was the resistance source. The genomic region controlling rust and LLS was successfully transferred in three elite and popular cultivars [13]. The additional achievement of this study was bringing together early maturity and disease resistance with significantly yield increase over their recurrent parents [14]. The breeding line NC94022 [15] has high level of resistance to TSWV [16] and therefore was used for development of the mapping population (named the S-population) for the identification of QTLs linked to this resistance trait. The first genetic map of the S-population had 172 marker loci spanning a total map distance of 920.7 cM [15] which was then improved to 206 mapped loci [17] with a total map distance of 1780.6 cM. In this study, this map was saturated and used in identification of QTLs associated with resistance to TSWV and leaf spots (early and late) and the markers will be validated for marker-assisted breeding selection in order to transfer the resistance trait(s) to elite peanut cultivars.

## Results

### Construction of improved genetic map

An improved genetic map with 248 marker loci was developed for the S-population using genotyping data of 258 marker loci (S1 Fig). The 248 markers were distributed onto 21 linkage groups (LGs) spanning a total map length of 1425.9 cM with density of 5.7 cM per loci (Table 1). The mapped marker loci per LG varied from three (B09 and LG21) to 25 (A03) with the mean of 12 loci per LG. Of the 248 mapped marker loci, 148 loci were mapped onto the 10 LGs of the A sub-genome with a total map distance of 831.8 cM and 97 marker loci mapped onto the 10 LGs of the B sub-genome with total map distance of 584.3 cM. Three marker loci without linkage to any LG of A or B sub-genomes were grouped to LG21 with a length of 9.8 cM (Table 1).

**Table 1:** Map features of the saturated genetic linkage map with 248 mapped loci of S-population.

Linkage Group	Mapped Loci	Length of LG (cM)	Map Density*	Linkage Group	Mapped Loci	Length of LG (cM)	Map Density
A sub-genome linkage group				B sub-genome linkage group			
A01	17	67.7	4.0	B01	18	71.1	4.0
A02	4	24.4	6.1	B02	8	46.0	5.7
A03	25	122.2	4.9	B03	16	99.4	6.2
A04	10	69.2	6.9	B04	15	75.0	5.0
A05	24	143.2	6.0	B05	4	49.1	12.3
A06	12	125.1	10.4	B06	7	65.0	9.3
A07	24	97.2	4.0	B07	8	76.0	9.5
A08	6	90.7	15.1	B08	12	35.0	2.9
A09	17	47.7	2.8	B09	3	43.7	14.6
A10	9	44.5	4.9	B10	6	23.9	4.0
*A-genome*	*148*	*831*.*8*	*5*.*6*	*B-genome*	*97*	*584*.*3*	*6*.*0*
additional linkage group	3	9.8	3.3	Total	248	1425.9	5.7

* Map density: average distance between two markers in terms of cM.

### Collinearity between genetic map and the physical map

**Fig 1:**
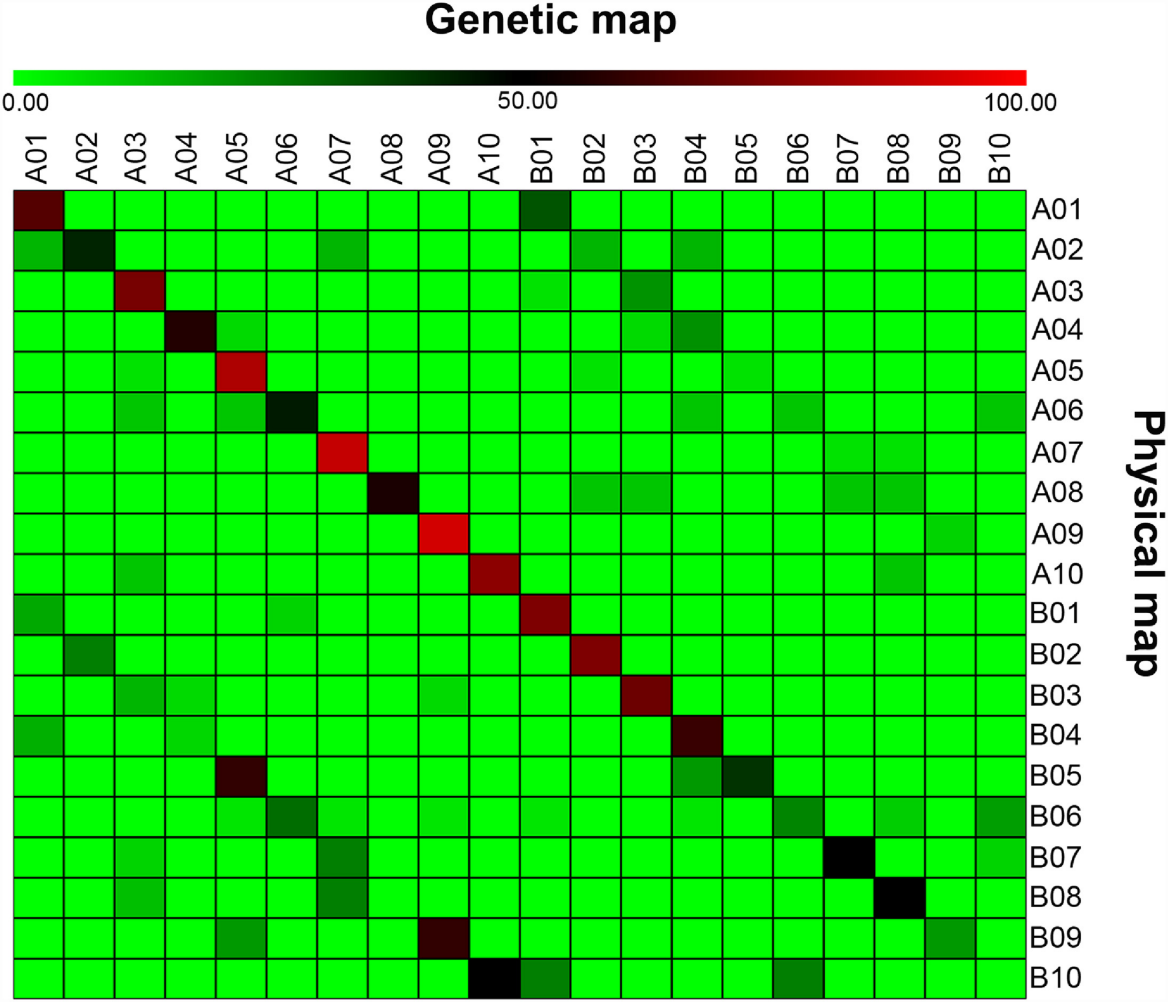
Comparison of markers mapped on different linkage groups (A01 to A10 and B01 to B10) of the genetic map of the S-population with the physical maps of two diploid peanut ancestors. The physical map of chromosomal pseudomolecules A01 to A10 of physical map of A sub-genome are from *Arachis duranensis* while that the B sub-genome B01 to B10, from *A*. *ipaensis*. Gradient color from light green to red denotes similarity percentage between the markers location in genetic map with that of physical map. The light green denotes 0% similarity, i.e. no common marker between the linkage group and the pseudomolecule, while red denotes 100% similarity, i.e. all the markers on one linkage group of genetic map were also present on the same pseudomolecule of physical map.

**Fig 2:**
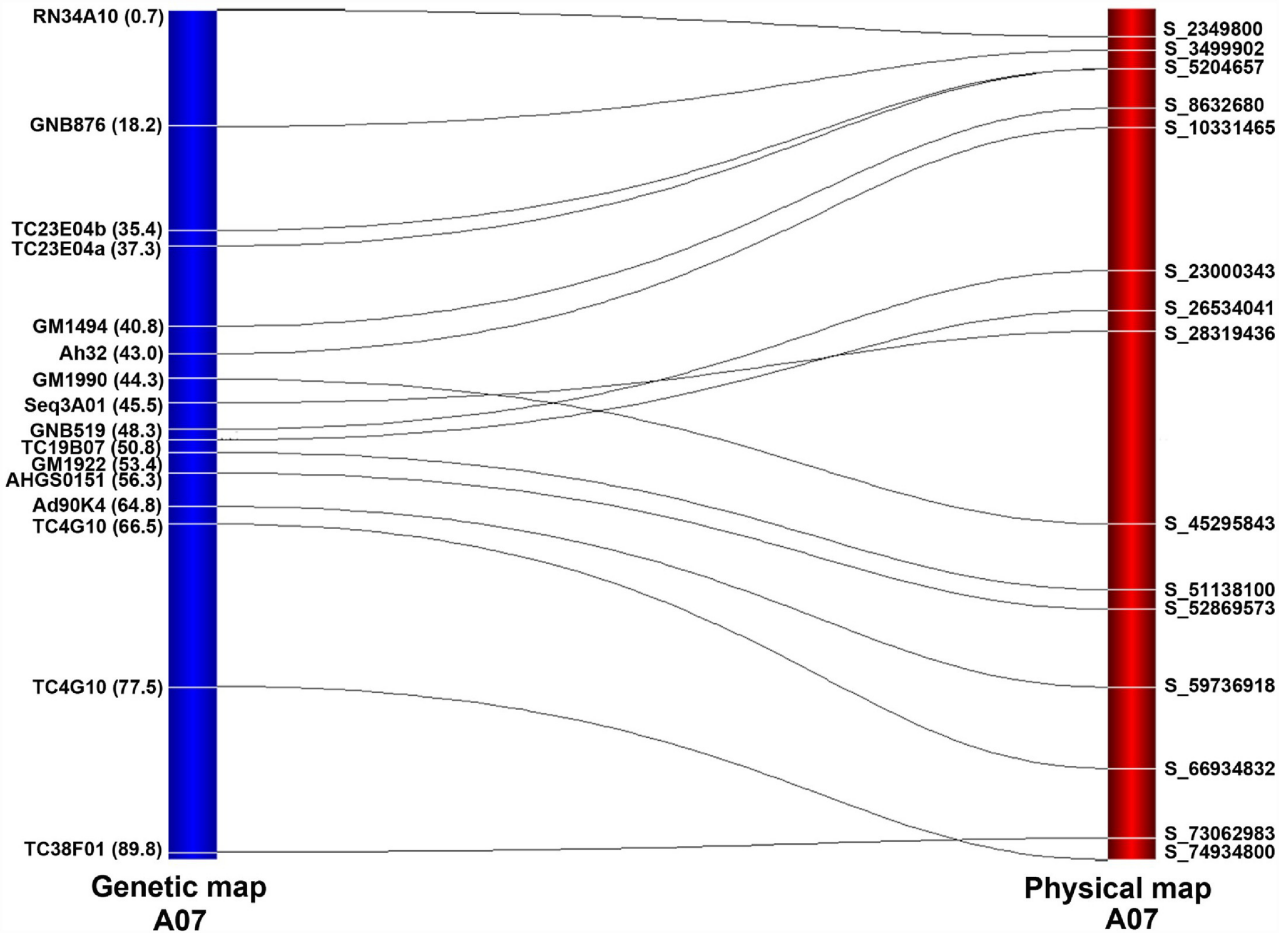
Collinearity of markers mapped on linkage group A07 from genetic map of the S population with the pseudomolecule of physical map. The lines connecting the two maps indicate the position of markers on genetic map with that of its relative position on physical map.

The availability of reference genomes for both the progenitors has allowed studying collinearity between genetic and physical map [18]. The available expressed sequence tag (EST) or genomic sequences of 233 SSR markers in public database were used for checking the level of collinearity between genetic and physical map. Of the 233 sequences, 229 could be uniquely aligned to different pseudomolecules of the A and B sub-genomes. Further, of these 229 aligned sequences, 131 sequences were placed on the A-genome and 98 sequences on the B-genome (S1 Table; Fig 1). In the case of the A-genome, a maximum of 19 sequences were aligned onto LG ‘A03’ and ‘A05’ while a minimum of seven sequences were aligned onto LG ‘A02’ with an average value of 13.1 sequences per LG. For the B-genome, the number of aligned sequences per LG ranged from four (B02 and B10) to 19 (B06) with an average value of 10 sequences per LG. Overall, about 60% collinearity was observed between the genetic map and the physical map. The average collinearity for the A sub-genome (68.29%) was higher than the B sub-genome (46.68%). Individually, maximum 91.67% collinearity was observed for LG ‘A09’, while markers from LG ‘B10’ showed no collinearity (Fig 1). A representative figure showing the collinearity for LG ‘A07’ is given in Fig 2 and for remaining LGs with more than five marker loci in S2 Fig.

### QTLs for disease resistance traits

**Fig 3:**
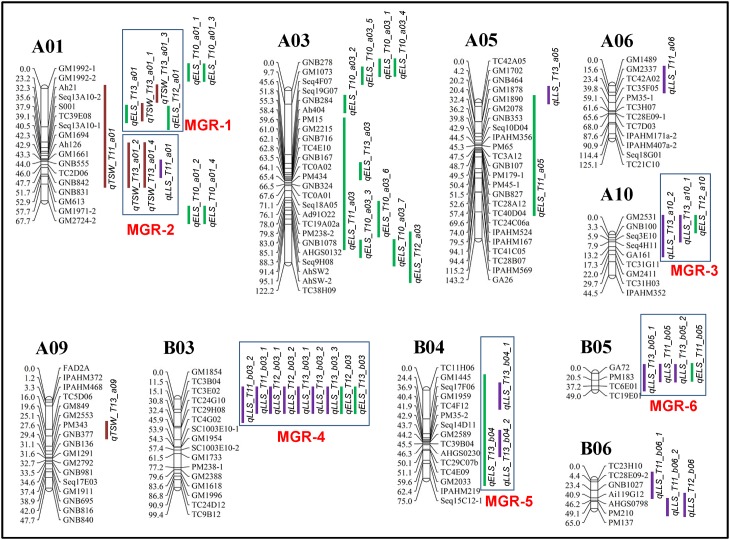
Genetic map of the S-population from the cross SunOleic 97R and NC94022 showing genomic regions harboring 48 QTLs for late leaf spot, early leaf spot and Tomato spotted wilt virus. A total of six major genomic regions (MGR) were found to have QTLs controlling more than one disease.

The quantitative trait locus (QTL) analysis conducted using multi-season phenotypic data and the improved genetic map resulted in the identification of 48 QTLs for three diseases in the S-population (Table 2). These QTLs were mapped onto 10 LGs (Fig 3) with percentages of phenotypic variation explained (PVE%) ranging from 3.88% (*qELS_T12_A01*) to 29.14% (*qTSW_T13_A01_4*) (S2 Table). Of the 48 QTLs, 16 QTLs had PVE larger than 10%, which were designated as major effect QTLs (Table 3). The distribution of the 48 QTLs across 10 LGs revealed that 29 QTLs were distributed across six LGs of the A sub-genome while 19 QTLs were distributed across four LGs of the B sub-genome. In the A sub-genome, 12 QTLs were identified on LG ‘A01’, 10 QTLs on LG ‘A03’, two QTLs on LG ‘A05’ while one QTL each was identified on LG ‘A06’ and ‘A09’. In the B sub-genome, nine QTLs were identified on LG ‘B03’, four on LG ‘B05’, and three QTLs on each LG ‘B04’ and ‘B06’.

**Table 2:** Summary of QTLs identified in the S-population for resistance to TSWV, ELS and LLS.

Trait / Year	Month of Observation	QTLs Identified	Major QTLs	LOD Value Range	Phenotypic Variance Explained (PVE%)	Additive Effect (a0)
*Tomato spotted wilt virus (TSWV)*						
2011	July	1	1	13	18.14	-0.39
2013	July	3	1	3.15–12.52	4.36–19.22	-0.22 to -0.46
2013	August	2	2	13.39–21.36	14.68–29.14	-0.57 to -0.63
*Early leaf spot (ELS)*						
2010	September	5	1	3.35–11.61	3.97–14.80	-0.57 to 0.80
2010	October	6	2	3.09–12.22	4.53–15.16	-0.64 to 1.17
2011	September	3	1	3.12–8.37	4.10–10.97	-0.21 to 0.23
2012	September	4	1	3.11–11.65	3.88–15.43	-0.18 to -0.37
2013	July	4	1	3.02–6.25	4.41–13.30	-0.09 to 0.17
*Late leaf spot (LLS)*						
2011	September	3	1	3.65–9.30	5.86–15.53	-0.34 to 0.27
2011	October	4	1	3.02–11.54	3.90–15.19	-0.32 to 0.16
2012	August	2	1	4.18–9.55	5.50–16.88	-0.36 to 0.20
2012	September	1	-	5.1	7.68	-0.5
2013	July	4	-	3.11–3.76	5.06–6.27	-0.13 to 0.13
2013	August	3	2	3.89–9.10	5.36–13.45	-0.30 to 0.27
2013	September	3	1	3.47–10.39	5.31–13.47	-0.38 to 0.24
Total		48	16	3.02–21.36	3.88–29.14	-0.64 to 1.17

**Table 3:** Details of the major effect QTLs identified for TSWV, ELS and LLS resistance in S-population.

S No.	QTL Name*	Year	Month	Linkage Group	Nearest Marker	Marker Interval	LOD Value	Phenotypic Variance Explained (PVE%)	Additive Effect (a0)
***Tomato spotted wilt virus (TSWV)***									
1	*qTSW_T11_A01*	2011	July	A01	TC39E08	Ah21-GNB842	13.01	18.14	-0.393
2	*qTSW_T13_A01_2*	2013	July	A01	GNB555	Ah126-GNB842	12.52	19.22	-0.466
3	*qTSW_T13_A01_3*	2013	July	A01	TC39E08	Ah21-Seq13A10-1	13.39	14.69	-0.577
4	*qTSW_T13_A01_4*	2013	August	A01	GNB555	Ah126-GNB842	21.37	29.14	-0.639
***Early leaf spot (ELS)***									
5	*qELS_T10_A01_2*	2010	September	A01	GM1971-2	GM2724-2-GM1971-2	11.62	14.80	-1.090
6	*qELS_T10_A01_4*	2010	October	A01	GM1971-2	GM2724-2-GM1971-2	12.23	15.16	1.174
7	*qELS_T10_A03_5*	2010	October	A03	GM1073	Seq4F07-GM1073	3.57	10.57	-0.966
8	*qELS_T11_A05*	2011	September	A05	PM65	GM1890-TC40D04	8.37	10.98	0.235
9	*qELS_T12_B03*	2012	September	B03	TC24G10	TC3E02-TC29H08	11.65	15.43	-0.377
10	*qELS_T13_B04*	2013	July	B04	GM1445	GM1445-GM2033	6.25	13.31	0.177
***Late leaf spot (LLS)***									
11	*qLLS_T11_B03_1*	2011	September	B03	TC3E02	TC3E02-TC29H08	9.31	15.53	-0.347
12	*qLLS_T11_B03_2*	2011	October	B03	TC29H08	TC3E02-TC4G02	11.55	15.20	-0.322
13	*qLLS_T12_A03_1*	2012	August	B03	TC3E02	TC3E02-TC29H08	9.56	16.89	-0.362
14	*qLLS_T13_B03_2*	2013	August	B03	TC3E02	TC3E02-TC29H08	9.10	13.46	-0.306
15	*qLLS_T13_B05_1*	2013	August	B05	PM183	GA72-TC6E01	4.85	10.59	0.271
16	*qLLS_T13_B03_3*	2013	September	B03	TC24G10	TC3E02-TC29H08	10.40	13.47	-0.388

* All the QTLs were identified from the phenotyping data generated at Tifton and the QTL nomenclature consisted of the letters as described that specified the “q” for QTL, the disease name “TSWV, ELS, or LLS”, the location “T” as Tifton, the numbers “10, 11, 12, or 13” for the year of 2010 to 2013, the sub-genome “A or B” for A or B followed by the number of the linkage group of “01 to 10”.

#### QTLs for Tomato spotted wilt virus (TSWV) resistance

Field evaluation experiments for disease resistance to TSWV in the years 2010, 2011 and 2013 had high disease incidence and severity and thus, good quality phenotyping data was available for further genetic analysis. The QTL analysis identified six QTLs for TSWV resistance with PVE ranging from 4.36% (*qTSW_T13_A09*) to 29.14% (*qTSW_T13_A01_4*) (Table 4). Interestingly, all six QTLs were distributed only on two LGs of the A-sub genome i.e., five QTLs on ‘A01’ and one QTL on ‘A09’ (Table 4). All the TSWV resistance alleles at the QTLs were contributed by the parent NC94022 (Table 4). Of these six QTLs, four QTLs were major effect QTLs with PVE ranging from 14.69% (*qTSW_T13_A01_3*) to 29.14% (*qTSW_T13_A01_4)* and were mapped on single LG i.e., ‘A01’ (Table 3).

**Table 4:** QTLs identified for Tomato spotted wilt virus (TSWV) resistance in S-population.

S No.	QTL Name	Year*	Linkage Group	Nearest Marker	Marker Interval	LOD Value	Phenotypic Variance Explained (PVE%)	Additive Effect (a0)
1	*qTSW_T11_A01*	2011	A01	TC39E08	Ah21-GNB842	13.01	18.14	-0.393
2	*qTSW_T13_A01_1*	2013	A01	S001	Ah21-TC39E08	4.71	6.28	-0.337
3	*qTSW_T13_A01_2*	2013	A01	GNB555	Ah126-GNB842	12.52	19.22	-0.466
4	*qTSW_T13_A09*	2013	A09	PM343	PM343-GNB377	3.15	4.36	-0.221
5	*qTSW_T13_A01_3*	2013	A01	TC39E08	Ah21-Seq13A10-1	13.39	14.69	-0.577
6	*qTSW_T13_A01_4*	2013	A01	GNB555	Ah126-GNB842	21.37	29.14	-0.639

* All the QTLs were identified from the phenotyping data generated at Tifton and observation recorded in the month of July at Tifton location.

#### QTLs for early leaf spot (ELS) resistance

For ELS, 22 QTLs were identified with PVE ranging from 3.88% (*qELS_T12_A01*) to 15.43% (*qELS_T12_B03*) (Table 5). Predominantly ELS was observed in the year 2010 while ELS was always present with LLS in the field from 2011 to 2013. The 22 QTLs were spread across seven LGs with the majority of 18 QTLs on four LGs of the A sub-genome and four QTLs on three LGs of the B sub-genome (Table 5). The maximum number of QTLs (10 QTLs) were mapped on LG ‘A03’ followed by 6 QTLs on LG ‘A01’. Six genomic regions could be identified for ELS i.e., three on LG ‘A01’, two on ‘A03’ and one on ‘B03’ (Table 5). A general terminology was used to term a QTL as a ‘consistent QTL’ when a particular QTL was observed at more than one field observation. Following above criteria, only two consistent QTLs were identified i.e., one on LG ‘A01’ between markers S001 to Seq13A10-1 and one on ‘B03’ between markers TC3E02 and TC4G02 (Fig 3, Table 5). Of the 22 QTLs, 15 QTLs had higher disease resistance ratings or scores from the parent SunOleic 97R indicating that low disease score was contributed by the parent NC94022. Of these 22 QTLs, six QTLs were major effect QTLs with PVE ranging from 10.57% (*qELS_T10_A03_5*) to 15.43% (*qELS_T12_B03)* (Table 3). Furthermore, two of the six *M-QTLs* were detected on LG ‘A01’.

**Table 5:** QTLs identified for early leaf spot (ELS) resistance in S-population.

S No.	QTL Name	Year*	Month of Observation	Linkage Group	Nearest Marker	Marker Interval	LOD Value	Phenotypic Variance Explained (PVE%)	Additive Effect (a0)
1	*qELS_T10_A01_1*	2010	September	A01	GM1992-1	GM1992-1-GM1992-2	3.78	4.40	0.592
2	*qELS_T10_A01_2*	2010	September	A01	GM1971-2	GM2724-2-GM1971-2	11.62	14.80	-1.090
3	*qELS_T10_A03_1*	2010	September	A03	GNB278	GNB278-GM1073	3.90	8.77	0.808
4	*qELS_T10_A03_2*	2010	September	A03	Ah404	GNB284-Ah404	3.35	3.97	-0.576
5	*qELS_T10_A03_3*	2010	September	A03	AHGS0132	GNB1078-AHGS0132	4.11	4.79	0.639
6	*qELS_T10_A01_3*	2010	October	A01	GM1992-1	GM1992-1-GM1992-2	4.00	4.53	-0.641
7	*qELS_T10_A01_4*	2010	October	A01	GM1971-2	GM2724-2-GM1971-2	12.23	15.16	1.174
8	*qELS_T10_A03_4*	2010	October	A03	GNB278	GNB278-GM1073	3.09	6.43	-0.738
9	*qELS_T10_A03_5*	2010	October	A03	GM1073	Seq4F07-GM1073	3.57	10.57	-0.966
10	*qELS_T10_A03_6*	2010	October	A03	Seq18A05	Seq18A05-PM238-2	3.53	5.94	-0.807
11	*qELS_T10_A03_7*	2010	October	A03	GNB1078	GNB1078-Seq9H08	5.41	6.47	-0.793
12	*qELS_T11_A03*	2011	September	A03	Seq18A05	PM15-GNB1078	7.05	9.19	-0.213
13	*qELS_T11_A05*	2011	September	A05	PM65	GM1890-TC40D04	8.37	10.98	0.235
14	*qELS_T11_B05*	2011	September	B05	GA72	GA72-PM183	3.13	4.10	0.142
15	*qELS_T12_A01*	2012	September	A01	Seq13A10-2	S001-Seq13A10-2	3.12	3.88	-0.188
16	*qELS_T12_A03*	2012	September	A03	PM238-2	PM238-2-AhSW2	4.04	5.22	-0.218
17	*qELS_T12_A10*	2012	September	A10	GM2531	GM2531-GNB100	4.59	5.89	-0.231
18	*qELS_T12_B03*	2012	September	B03	TC24G10	TC3E02-TC29H08	11.65	15.43	-0.377
19	*qELS_T13_A01*	2013	July	A01	TC39E08	S001-TC39E08	3.03	4.12	-0.098
20	*qELS_T13_A03*	2013	July	A03	TC0A02	TC0A02-PM434	3.17	4.61	-0.103
21	*qELS_T13_B03*	2013	July	B03	TC3E02	TC3E02-TC29H08	4.44	7.76	-0.135
22	*qELS_T13_B04*	2013	July	B04	GM1445	GM1445-GM2033	6.25	13.31	0.177

* All the QTLs were identified from the phenotyping data generated at Tifton location.

#### QTLs for late leaf spot (LLS) resistance

Late leaf spot (LLS) was predominantly observed during the years 2011 to 2013. QTL analysis resulted in the identification of 20 QTLs with PVE ranging from 3.90% (*qLLS_T11_B06_2*) to 16.89% (*qLLS_T12_B03_1*) (Table 6). The 20 QTLs were distributed on eight LGs with seven QTLs located on LG ‘B03’. However, there were 3 genomic regions each on three LGs (B03, B05 and B06) wherein 13 QTLs were congregated (Table 6). On LG ‘B03’ (TC3E02 to TC29H08), LLS resistance was contributed by the parent NC94022 for all the 7 QTLs (Table 6). On the contrary, in the LGs ‘B05’ (GA72 to PM183) and ‘B06’ (TC28E09-2 to PM210), the LLS resistance was contributed by the parent SunOleic 97R. Of these 20 QTLs, six QTLs were major effect QTLs with PVE ranging from 10.59% (*qLLS_T13_B05_1*) to 16.89% (*qLLS_T13_B05_1)* (Table 3). Furthermore, five of the six *M-QTLs* were detected on LG ‘B03’.

**Table 6:** QTLs identified for late leaf spot (LLS) resistance in S-population.

S No.	QTL Name	Year*	Month of Observation	Linkage Group	Nearest Marker	Marker Interval	LOD Value	Phenotypic Variance Explained (PVE%)	Additive Effect (a0)
1	*qLLS_T11_B03_1*	2011	September	B03	TC3E02	TC3E02-TC29H08	9.31	15.53	-0.347
2	*qLLS_T11_B05*	2011	September	B05	GA72	GA72-PM183	3.66	5.86	0.213
3	*qLLS_T11_B06_1*	2011	September	B06	GNB1027	TC28E09-2-Ai119G12	4.83	9.56	0.272
4	*qLLS_T11_A01*	2011	October	A01	TC2D06	GNB555-TC2D06	3.37	4.07	-0.166
5	*qLLS_T11_A06*	2011	October	A06	GM2337	GM2337-TC35F05	3.28	4.39	-0.173
6	*qLLS_T11_B03_2*	2011	October	B03	TC29H08	TC3E02-TC4G02	11.55	15.20	-0.322
7	*qLLS_T11_B06_2*	2011	October	B06	AHGS0798	AHGS0798-PM210	3.02	3.90	0.164
8	*qLLS_T12_B03_1*	2012	August	B03	TC3E02	TC3E02-TC29H08	9.56	16.89	-0.362
9	*qLLS_T12_B06*	2012	August	B06	PM210	Ai119G12-PM210	4.18	5.51	0.207
10	*qLLS_T12_B03_2*	2012	September	B03	TC29H08	TC3E02-TC29H08	5.11	7.68	-0.510
11	*qLLS_T13_A05*	2013	July	A05	GM1878	GM1878-GM1890	3.12	5.86	0.135
12	*qLLS_T13_B03_1*	2013	July	B03	TC3E02	TC3E02-TC29H08	3.52	6.28	-0.137
13	*qLLS_T13_B04_1*	2013	July	B04	Seq17F06	TC4F12-Seq17F06	3.77	5.97	0.136
14	*qLLS_T13_B04_2*	2013	July	B04	AHGS0230	GM2589-AHGS0230	3.59	5.07	0.128
15	*qLLS_T13_A10_1*	2013	August	A10	GNB100	GM2531-Seq3E10	3.89	5.37	-0.192
16	*qLLS_T13_B03_2*	2013	August	B03	TC3E02	TC3E02-TC29H08	9.10	13.46	-0.306
17	*qLLS_T13_B05_1*	2013	August	B05	PM183	GA72-TC6E01	4.85	10.59	0.271
18	*qLLS_T13_A10_2*	2013	September	A10	GNB100	GM2531-GA161	6.64	8.39	-0.303
19	*qLLS_T13_B03_3*	2013	September	B03	TC24G10	TC3E02-TC29H08	10.40	13.47	-0.388
20	*qLLS_T13_B05_2*	2013	September	B05	GA72	GA72-PM183	3.47	5.31	0.241

* All the QTLs were identified from the phenotyping data generated at Tifton location.

### Gene-rich regions with multiple disease resistance QTLs

If genomic regions harbor QTLs which are associated with resistance to at least two of the three diseases, then these regions were termed as major genomic regions (MGR) in the present study. Of the 48 QTLs detected, 44 were clustered in 11 genomic regions across seven LGs. However, MGRs were only found in six genomic regions across five LGs (Fig 3). In LG ‘A01’, two MGRs were found (MGR-1 and MGR-2). In MGR-1 (Ah21 to Seq13A10-1), four QTLs were identified, two each for ELS and TSWV with resistance alleles from the parent NC94022. In the genomic region MGR-2 (Ah126 to GNB842), three QTLs were present, two QTLs for TSWV and one for LLS with all resistance alleles from the parent NC94022.

The genomic region MGR-3 on LG ‘A10’ (GM2531 to GA161) had three QTLs, two for LLS and one for ELS. All the three resistance alleles were contributed by the parent NC94022. The MGR-4 (TC3E02 to TC4G02) on LG ‘B03’ had nine QTLs, seven for LLS and two for ELS with PVE ranging from 6.28% to 16.89%. In this region, all the resistance alleles were contributed by NC94022. The MGR-5, located on LG ‘B04’ (GM1445-GM2033), harbored three QTLs, two for LLS and one for ELS with PVE ranging from 5.07% to 13.31%. Lastly, in MGR-6 (GA72 to TC6E01) on LG ‘B05’, there were four QTLs, i.e., three for LLS and one for ELS with PVE ranging from 4.10% to 10.59%. Interestingly, resistance alleles in both MGR-5 and MGR-6 were contributed by the parent SunOleic 97R (Fig 3).

## Discussion

Majority of the peanut varieties cultivated globally are susceptible to a range of diseases including TSWV, ELS and LLS. These three diseases cause significant yield loss globally in addition to putting extra financial burden on farmers by investing in costly chemical-based disease control. The most promising strategy for managing the damages caused by diseases is the development of resistant varieties and their cultivation in farmers’ field of disease prone areas of the world. Molecular markers linked to the resistance for important diseases could be deployed to breed resistant varieties. In this context, an appropriate genetic mapping population with accurate phenotypic data is needed to develop a dense genetic map for identification of consistent and stable QTLs. The objective of the current study was to identify genomic regions closely associated with disease resistance traits in peanut, which could then be used in genetic improvement of peanut cultivars with desired disease resistance trait(s) through genomics-assisted breeding (GAB).

### Importance of precise phenotyping

Precise and accurate phenotyping is necessary for establishing a reliable marker-phenotype association. In general, phenotyping for disease resistance is done using measurable quantities of inoculum and disease incidence in a greenhouse. Many times, QTLs identified from greenhouse experiments have higher PVE, however this PVE is reduced drastically when the same population is tested under field conditions. For cultivar development, it is necessary that the QTL expresses consistently in a range of field conditions. In this study, phenotyping in the field conditions for three diseases of peanut namely TSWV, ELS and LLS were conducted from 2010 to 2013 in Tifton, Georgia, with two planting dates (April and May) each year to insure higher disease incidences. The first planting in April was better for scoring TSWV incidences, while the second planting in May was better for ELS and LLS ratings with less influence of TSWV. Multiple readings were taken during the growing season so that disease ratings could be confirmed. Subsequently, the genomic regions containing QTLs from multiple readings of different diseases were identified as “MGR”. Disease development varied from time to time and year to year and the diverse patterns of incidences were affected by environment. For example, ELS was dominant in the year 2010, however moderate incidence of ELS was observed during 2011, 2012 and 2013. In contrast, high incidence of LLS was observed during the cropping seasons of 2011 to 2013. Heavy TSWV incidences were only observed in 2010 and 2013. It was evident from the results that only two QTLs for ELS could be detected in 2011 when the disease incidence was low, while 11 QTLs were identified during the year 2010 when the incidence was high.

### Use of dense genetic map and collinearity

In order to realize the full potential of phenotyping, it is necessary to have a highly dense genetic map. In the present study, the genetic map of the S-population was saturated to 248 marker loci on 21 LGs (A01 to A10, B01 to B10, and LG21) with total length of 1425.9 cM. A comparatively high number of marker loci was found in the A sub-genome (148) as compared to the B sub-genome (97) indicating that the A sub-genome contains higher diversity as compared to the B sub-genome.

RIL populations have several unique advantages over F_2_ and backcross populations, such as the possibility to generate multi-location and multi-year phenotyping data. Recently, several RIL populations of peanut were developed and used for development of SSR-based genetic maps. Varshney et al. [19] reported the first SSR-based genetic map for peanut, with 135 marker loci which was further saturated to 191 loci by Ravi et al. [20] in the RIL population TAG 24 × ICGV 86031. In the two RIL populations from the cross TAG 24 × GPBD 4 and TG 26 × GPBD 4, marker loci were increased to 188 and 181 marker loci, respectively [12] from initially 56 and 45 marker loci, respectively [11, 21]. In the US, we have developed a RIL population (S-population) which was initially used to develop a genetic map with 172 marker loci and a map distance of 920.7 cM [15], which was then improved to 206 marker loci with a map distance of 1780.6 cM [17]. Another RIL population named the T-population was developed which initially had 236 mapped loci [15] and was later improved to 377 mapped loci [17].

The availability of peanut genome sequences has enabled comparative genomic studies. In the present study, we predicted the physical location of 229 SSR markers on the current reference genome assembly and thereby tried to redefine the earlier identified linkage groups. Out of 229, 57.2% of the sequences were aligned to the A-genome while 42.8% were aligned to the B-genome, indicating less similarity between these two genomes as reported earlier [22]. The physical and genetic location of each marker was also compared and showed fairly high (57.26%) collinearity. Interestingly, some markers whose physical location was predicted on one chromosome were found mapped on the respective homeologous chromosome, indicating synteny between the homeologous chromosomes. Guo et al. [23] also reported the presence of macrosyntenic regions between A and B sub-genomes. This study will help to determine the physical location of identified QTLs and further for candidate gene identification.

### Linkage groups harboring disease resistance genes

Marker-trait association for all the three diseases revealed a total of 48 QTLs, out of which 31.34% (16 QTLs) were major effect QTLs indicating that a few QTLs can partially cover the disease resistance trait. Looking at the genome-wise distribution of these QTLs, a majority (29 QTLs) were present in the A sub-genome and 19 QTLs were in the B sub-genome, indicating that A sub-genome is more rich in harboring resistance genes than the B sub-genome, as suggested by [18]. A total of 6 QTLs were identified for TSWV resistance, all located on the A sub-genome, with five QTLs on LG ‘A01’. In an earlier study by Qin et al. [15] using the S-population, one QTL (*qTSWV2*) was identified for TSWV on LG ‘A01’ and at a similar position of the current map. In another study, by Wang et al. [24], a total of 15 QTLs from an F_2_ map and nine QTLs from an F_5_ map were identified for TSWV in the T-population. The QTLs for resistance were spread on 10 linkage groups of the F_2_ map and on 14 linkage groups of the F_5_ map. Of these two maps, linkage groups I, II, V, VI, IX, XI, XII and XVIII were common for both the mapping populations and harbored majority of the QTLs for resistance [24].

For ELS, 22 QTLs were identified, of which 18 QTLs were present on the A sub-genome and four QTLs, on the B sub-genome. LG ‘A03’ had the maximum number of QTLs (10 QTLs) within three genomic regions in which two genomic regions had consistent QTLs. On the LG ‘B03’, one more consistent QTL was identified. Most of the alleles for disease resistance were contributed by the parental line NC94022. These consistent QTLs could be helpful for genomic-assisted breeding programs. This is the first study in peanut wherein QTLs specifically identified for ELS have been reported using a bi-parental mapping population. Nevertheless, Pandey et al. [25] reported six marker-trait associations (MTAs) for ELS with 9.18–10.99% PVE using a genome-wide association study (GWAS) mapping approach.

For LLS, a total of 20 QTLs were identified in which 15 QTLs were on the B sub-genome and seven QTLs were on LG ‘B03’. It seems that the B sub-genome harbors more LLS resistance as compared to the A sub-genome. In an earlier study from a F_2_ population of the cross *A*. *duranensis* ‘K7988’ and *A*. *stenosperma* ‘V10309’, a total of five QTLs were identified [26]. In another study, Sujay et al. [12] identified 13 major QTLs for resistance to LLS with PVE in the range of 10.27% to 67.98% in two RIL populations namely TAG 24 × GPBD 4 and TG 26 × GPBD 4, in which the resistance was contributed by the parental line GPBD 4. Different marker sets were used for constructing the genetic maps in both of these studies and the comparison between the maps was difficult for the commonality of genomic regions identified controlling disease resistance in these studies. Besides the bi-parental populations, Pandey et al. [25] reported one marker-trait association (MTA) for LLS with 18.1% PV using a GWAS approach.

### Genomic regions with plausible pleiotropic effect

Visualization of all the QTLs onto the genetic map revealed that out of the 48 QTLs, 44 QTLs were clustered onto 11 genomic regions of seven LGs. Interestingly, three LGs, A01, A03 and B03, had the most QTLs, with 10, 12 and nine QTLs, respectively. In most cases, the resistance alleles were contributed by the parental line NC94022, however, there were several QTLs from SunOleic 97R, the susceptible parent. This is a unique case and an opportunity to further study the disease resistance mechanism for the ELS, LLS and TSWV resistance in peanut. Therefore, the present study should be useful in helping to design an appropriate genomics-assisted breeding program to enhance disease resistance for all three diseases.

## Materials and Methods

### Mapping population

A recombinant inbred line (RIL) population was developed from the cross SunOleic 97R and NC94022 with 352 individuals at the Crop Protection and Management Research Unit of USDA-ARS, Tifton, GA [14] using the single seed descent method. The male parent NC94022 is a Virginia market type with high level of field resistance to TSWV [16]. It was derived from a cross between N91026E, an early maturing Virginia type line moderately susceptible to TSWV, and PI 576638, a *hirsuta* botanical type line from Mexico [27]. The female parent, SunOleic 97R is a runner market-type with high oleic acid content and is susceptible to TSWV [28]. ‘SunOleic 97R’ was a selection from the cross between F435-2-2-E-2-l-b4-E-b2-b3-l-E, a high oleic line with *FAD2A* and *FAD2B* genes and Sunrunner (F519-9), a runner market-type line [17, 28–31].

### Phenotyping for disease resistance

The RIL population with 352 individuals along with two parents were phenotyped for TSWV and leaf spots (LS) including both ELS and LLS. The field experiments were conducted from 2010 to 2013 in the Bellflower Farm of USDA-ARS, Crop Protection and Management Research Unit at Tifton, GA, using a randomized complete block design having at least three replications. There were no specific permissions required for this field experiments and these field activities did not involve endangered or protected species. The soil type was Tifton loamy sand having characteristics such as siliceous, fine-loamy and thermic Plinthic Kandiudult. The experimental plots were 3.0 m long, separated by an alley of 1.5 m. The seeds were planted to 91-cm-spaced single-row pattern with two rows per plot. Seeding rate was 10 seeds/m of row. Sparse seeding rate was used to maximize pressure of Tomato spotted wilt. In the current study, more emphasis was given to reaction of plants to field level disease resistance. There were two planting dates each year, April and May. Only April planted trails were used for the spotted wilt rating, and both April and May planting trails were used for evaluations of leaf spots (ELS and LLS) in order to have optimized disease ratings [32].

In order to ensure the disease severity ratings for all three diseases (TSWV, ELS and LLS), an attempt was made to record disease ratings or scores at multiple assessment dates during the trial period. Observations or readings (r) were designated based on the month when the observation was taken, such as r1 for July, r2 for August, r3 for September and r4 for October. During the trials from 2010 to 2013, a total of 23 readings were successfully taken for three disease ratings. The extent of disease incidence of TSWV was obtained following Culbreath et al. [33] and Baldessari [34] using a 0 to 5 disease intensity rating. Similarly the prevalence of LS (ELS and/or LLS) disease was evaluated on the scale of 1 to 10 as detailed in Chiteka et al. [35] and Wang et al. [24].

### DNA isolation and genotyping

The DNA was extracted from the fresh leaves of all the RILs along with the parental genotypes as described in Qin et al. [15]. The quantity and quality of the genomic DNA was evaluated using Nano-Drop 1000 spectrophotometer (Nano Drop Technologies, USA). PCR reactions were carried out in a 15 μl reaction volume using thermal cycler DNA Engine Tetrad 2 Peltier (BioRad Laboratories, Hercules, CA, USA) or PTC-225 DNA Engine Tetrad Peltier (MJ Research, Waltham, MA, USA). The PCR master mix was prepared using 25 ng genomic DNA, 0.5 μM of each primer, 10× PCR buffer, 0.2 mM dNTPs, 1.5 mM MgCl_2_ and 0.5 U *Taq* polymerase. The PCR reaction, the documentation of PCR profile and scoring of PCR bands was done as details provided in Qin et al. [13]. The PCR was performed for all the markers at the temperature profile of 95°C for 4 min, 35 cycles of 45 s at 94°C, 45 s at 55°C and 1 min at 72°C, and a final extension step of 7 min at 72°C. Amplified PCR products were visualized and scored on non-denaturing polyacrylamide gel (PAGE) at 160 V for 1 h 20 min, while electrophoresis gels was performed at 180 V for 1 h 40 min.

### Saturating the genetic map

The first genetic map of S-population had 172 marker loci [15] which was then improved to 206 mapped loci [17]. In the present study, an additional 52 polymorphic markers were identified and genotyped on the S-population. Genotyping data for all the 258 markers (206 marker loci from the genetic map of Pandey et al. [17] plus 52 new marker loci) were scored as “a” and “b” as per the format of JoinMap^®^ version 4 [36] for the construction of an improved genetic map. The function “locus genotype frequency” was used first to calculate chi-square values for analyzing segregation distortion for each marker loci against the expected 1:1 ratio. In order to accommodate the maximum number of marker loci in the genetic map, a genetic framework map was constructed with markers that were normally segregating at minimum logarithm of odds (LOD) of 3.0 (maximum LOD value 7.0) and maximum recombination at 25% using the command “LOD groupings” and “create groups for mapping” into respective linkage groups (LG) and map function Kosambi [37]. After developing the framework genetic map, the unmapped (distorted) markers were integrated into different linkage groups of the framework genetic map at recombination frequency up to 50%. The visualization of linkage maps was done using the software MapChart 2.2 [38].

### Determining physical position of markers in the sub-genomes

The international peanut genome initiative has sequenced the genomes of peanut progenitors, *A*. *duranensis* (A genome, 2n = 2x = 20) and *A*. *ipaensis* (B genome, 2n = 2x = 20) (www.peanutbase.org) [18]. The physical location of SSR markers used for mapping was predicted by aligning the EST or genomic sequence of each marker against a reference genome using the BLASTN program in two steps. Initially, the program was run with an e-value cutoff of 10^−25^. The top blast hit for each sequence was extracted and the physical position of EST sequence was determined based on the coordinates of subject hit. In the second step, for sequences which did not get any hit, BLASTN program was run with less stringency (e-value 10^−15^) and physical positions were determined as mentioned above. The collinearity was determined by comparing physical and genetic maps. The map order was visualized using Strudel V. 1.12.03.20 [39] while heat map showing percentage of collinearity was generated using MeV V4.9 [40].

### QTL analysis and visualization

The QTL mapping was done for all phenotyping readings recorded during 2010–2013 for three disease resistance traits. The Windows QTLCartographer version 2.5 [41] was used for the identification of QTLs. Various parameters for composite interval mapping (CIM) were used such as 1.0 cM as scanning interval between markers and tentative QTL with window size of 10.0, model 6, 500 permutations, and 0.05 significance level. The GGT 2.0 software [42] was used for visualizing the genotyping data and alleles for a marker in each line.

## Supporting Information

S1 Fig Genetic linkage map of the S-population from the cross SunOleic 97R and NC94022. This genetic map shows map location and order of 248 mapped loci on the 21 linkage groups.
(PDF)

S2 Fig Collinearity of mapped marker loci between genetic and physical map. The lines connecting the two maps indicate the position of markers on genetic map with that of its relative position on physical map.
(PDF)

S1 Table Collinearity between genetic and physical map. This table shows the collinearity between mapped loci in a linkage group and its corresponding pseudomolecule of A and B sub-genome.
(XLSX)

S2 Table Summary of main effect QTLs (M-QTLs) identified by QTLCartographer in S-population. This table has detailed information on each QTL detected for three diseases on season, location, linkage group, nearest marker, marker interval, LOD value, phenotypic variance explained, additive effect and source of contributing allele.
(XLSX)
