# Supplementary figures and images for: Mapping Quantitative Trait Loci of Resistance to Tomato Spotted Wilt Virus and Leaf Spots in a Recombinant Inbred Line Population of Peanut (Arachis hypogaea L.) from SunOleic 97R and NC94022
Source: PLoS One. 2016 Jul 18;11(7):e0158452. doi: 10.1371/journal.pone.0158452 (PMC4948827; doi:10.1371/journal.pone.0158452)

**A01**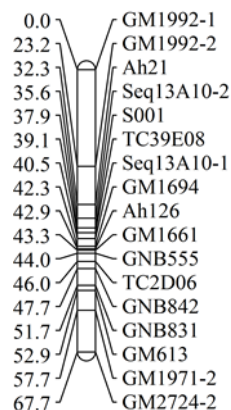**A02**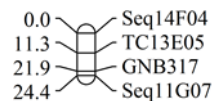**A03**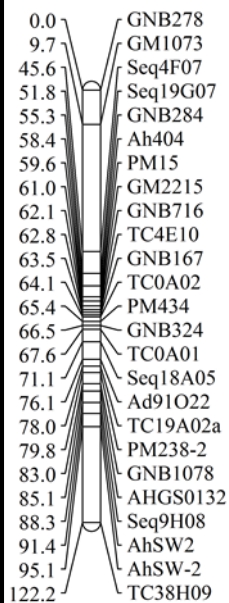**A04**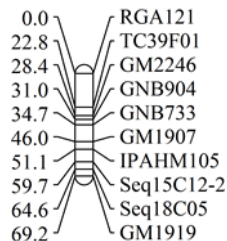**A05**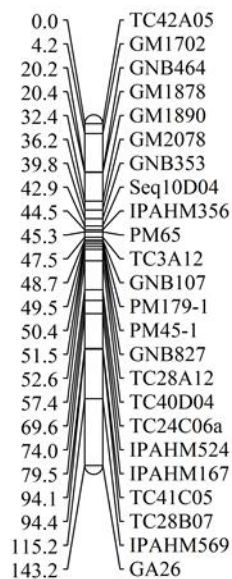**A06**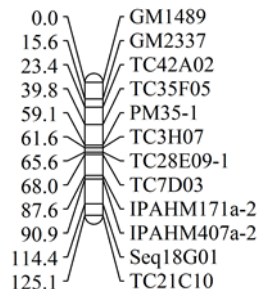**A07**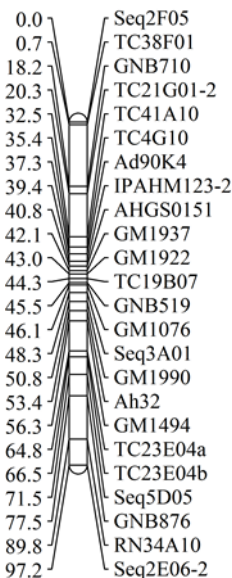**A08**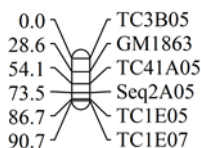**A09**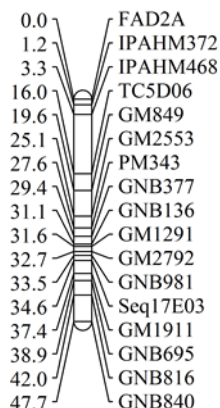**B01**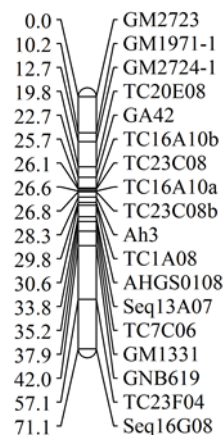**B02**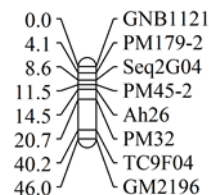**A10**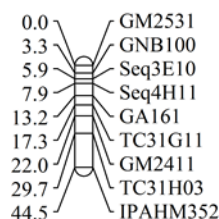**B03**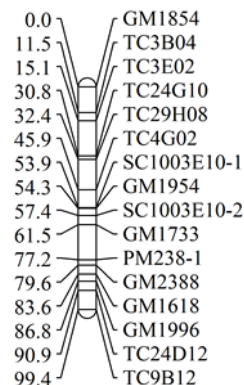**B04**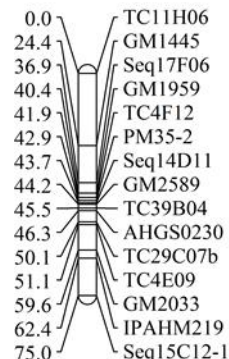**B05**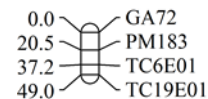**B06**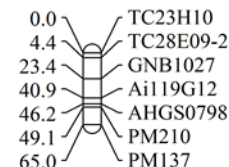**B07**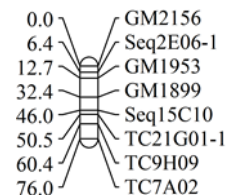**B08**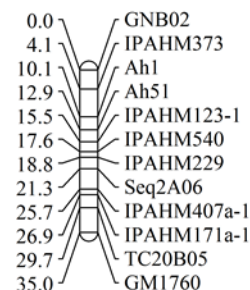**B09**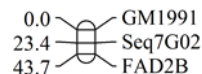**B10**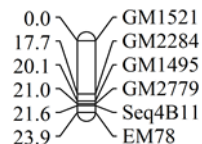**LG21**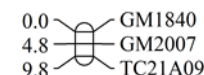

Supplement: S1 Fig — This genetic map shows map location and order of 248 mapped loci on the 21 linkage groups. (PDF) [file pone.0158452.s001.pdf]

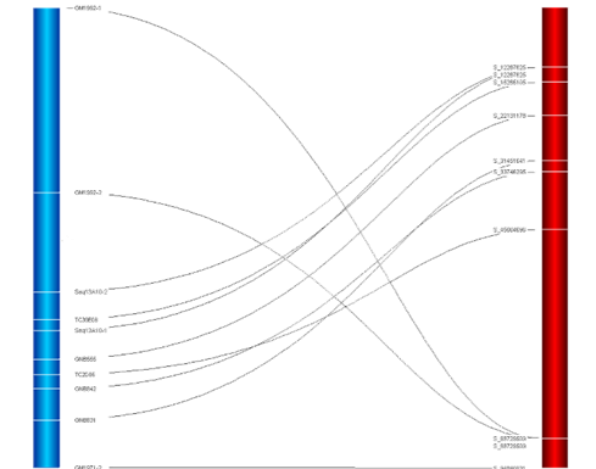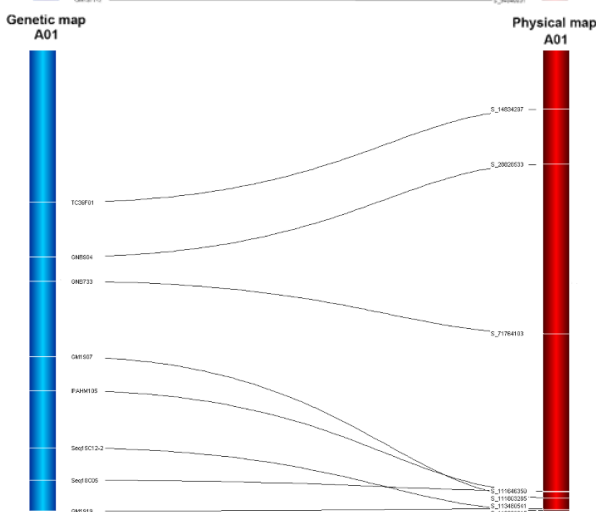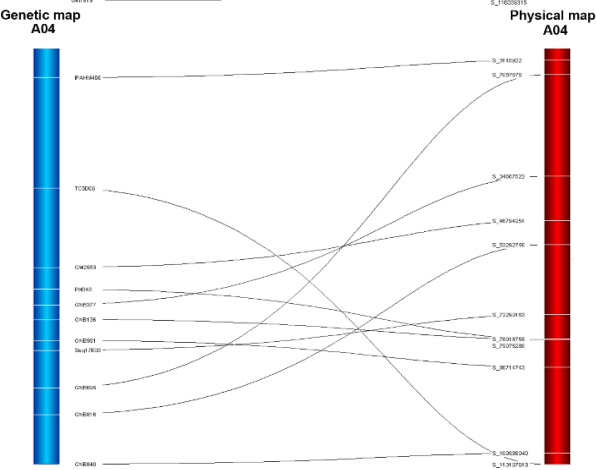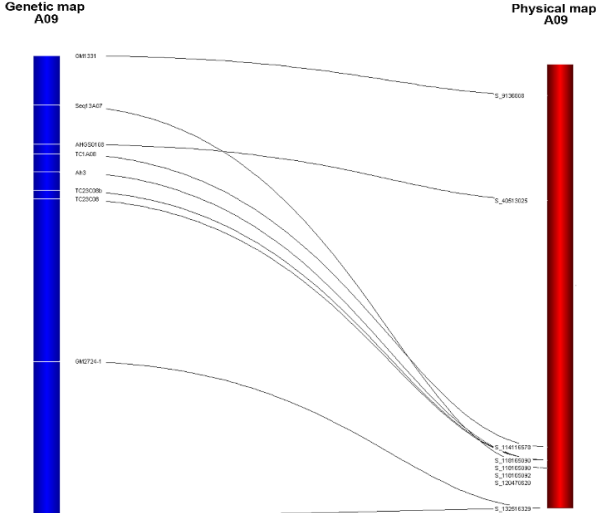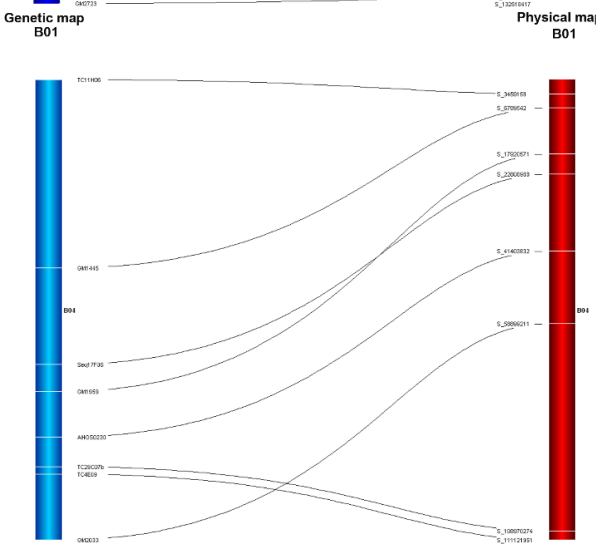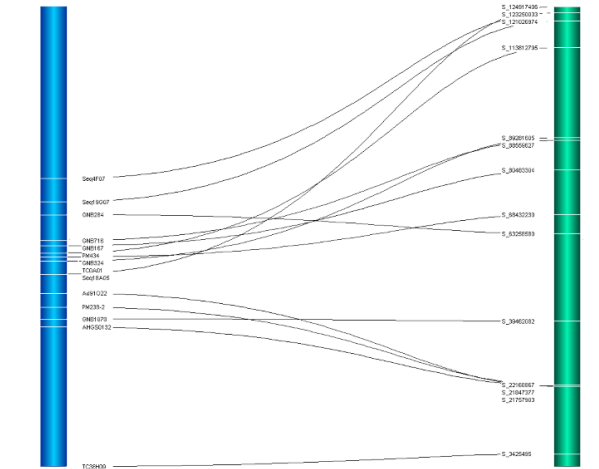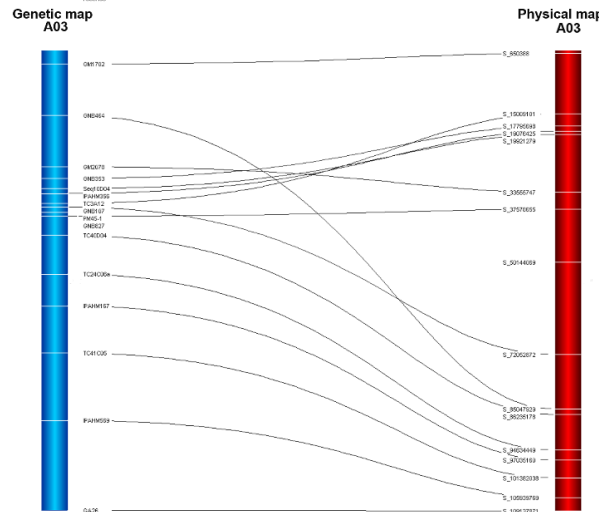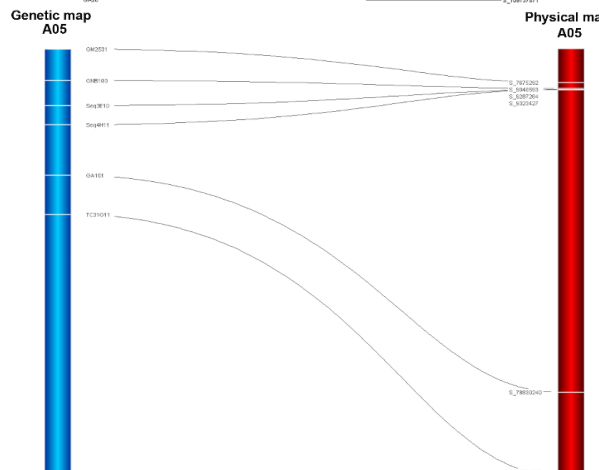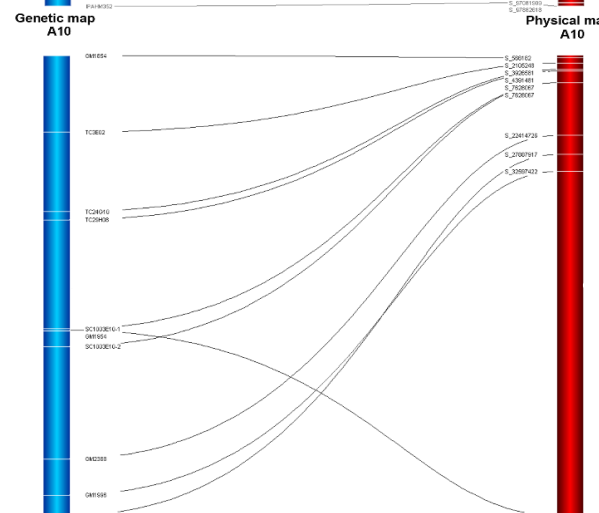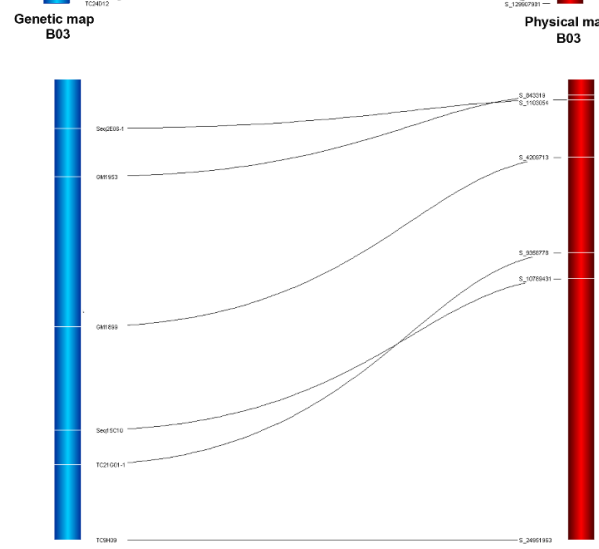

Supplement: S2 Fig — The lines connecting the two maps indicate the position of markers on genetic map with that of its relative position on physical map. (PDF) [file pone.0158452.s002.pdf]
